# Helminth-induced immune modulation in colorectal cancer: exploring therapeutic applications

**DOI:** 10.3389/fimmu.2025.1484686

**Published:** 2025-04-14

**Authors:** Hongyu Li, Chaojun Shan, Yunhuan Zhu, Xiaodong Yao, Lijun Lin, Xiaofen Zhang, Yuncheng Qian, Yuqing Wang, Jialu Xu, Yijie Zhang, Hairun Li, Ling Zhao, Keda Chen

**Affiliations:** ^1^ Key Laboratory of Artificial Organs and Computational Medicine of Zhejiang Province, Shulan International Medical College, Zhejiang Shuren University, Hangzhou, China; ^2^ Ocean College, Beibu Gulf University, Qinzhou, China; ^3^ School of Marxism, Hangzhou Medical College, Hangzhou, Zhejiang, China; ^4^ School of Basic Medicine and Forensic Medicine, Hangzhou Medical College, Hangzhou, China

**Keywords:** colorectal cancer, precursors of colorectal cancer, helminth therapy, helminth-derived products, immune modulation, combination therapy

## Abstract

Colorectal cancer is one of the most lethal tumors, posing a financial and healthcare burden. This study investigates how helminths and pre-existing diseases such as colitis, obesity, diabetes, and gut microbiota issues influence colon cancer development and prognosis. The immune system’s protective immunosuppressive response to helminth invasion minimizes inflammation-induced cell damage and DNA mutations, lowering the risk of colorectal cancer precursor lesions. Helminth infection-mediated immunosuppression can hasten colorectal cancer growth and metastasis, which is detrimental to patient outcomes. Some helminth derivatives can activate immune cells to attack cancer cells, making them potentially useful as colorectal cancer vaccines or therapies. This review also covers gene editing approaches. We discovered that using CRISPR/Cas9 to inhibit live helminths modulates miRNA, which limits tumor growth. We propose more multicenter studies into helminth therapy’s long-term effects and immune regulation pathways. We hope to treat colorectal cancer patients with helminth therapy and conventional cancer treatments in an integrative setting.

## Graphical Abstract

Graphical summary. The “Helminth infection” section demonstrates that the presence of helminths in the host's body leads to activating a sequence of immunological responses. This encompasses an elevation in pro-inflammatory molecules (IFN-α, IL-1β, IL-12) and the control of anti-inflammatory molecules (TGF-β, IFN-10). A well-regulated immune response can decrease cellular harm and DNA alterations resulting from inflammation, diminishing the likelihood of developing colorectal cancer precursor lesions. Nevertheless, the helminth infection's immunosuppressive environment may also facilitate the growth and spread of cancer, adversely affecting patient outcomes. The "Parasite antigens" section illustrates the mechanisms by which dendritic cells (DCs) internalize and digest parasite antigens, presenting them to CD8+ T lymphocytes. Activated T cells secrete cytotoxic chemicals such as PFN and GzmB, as well as cytokines like IFN-γ and TNF-α, which cause cancer cells to die. This approach proposes that antigens produced from helminths have the potential to be utilized as vaccines or therapies for colorectal cancer. This would include utilizing the immune system to specifically target and eliminate cancer cells. The section on “CRISPR/Cas9 gene editing helminth” focuses on the progress made in gene editing technologies, specifically CRISPR/Cas9, that enable accurate alterations to the helminth genome. Researchers can reduce the harmfulness of live helminths by selectively focusing on particular sequences in the genomic DNA. These genetically altered parasitic helminths can be employed to control the levels of microRNA, decrease the expression of cancer-promoting microRNAs, and stabilize the messenger RNA of tumor suppressor genes, so effectively impeding the formation of tumors.

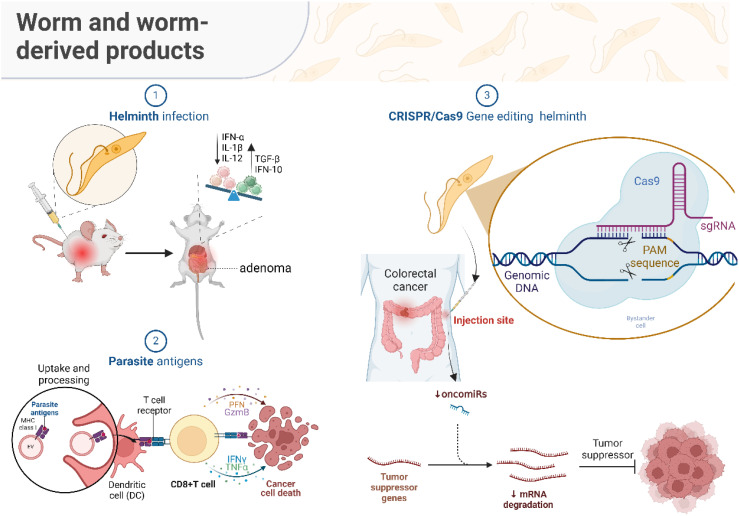

## 1 Introduction

Poor hygienic conditions mainly cause helminth infections, particularly in less developed socioeconomic and industrialized areas (1). A common feature of these helminths is their ability to survive long-term within the host (2). This requires helminths to possess efficient strategies to evade the host’s immune system attacks. Regardless of the type of helminth, they are rapidly detected by the immune system, triggering responses from stromal cells such as epithelial cells and keratinocytes, which secrete alarm protein molecules such as thymic stromal lymphopoietin, IL-25, and IL-33 (3). These proteins impact type 2 innate lymphoid cells (ILCs), which release cytokines including IL-5 and IL-13. In particular, IL-13 can interact with dendritic cells (DCs) to assist them in polarizing naïve T cells into a Th2 phenotype (4), suppressing pro-inflammatory reactions.

Colorectal cancer (CRC) is one of the leading tumors worldwide and, along with lung cancer, prostate cancer, and breast cancer, is considered a major human killer (5). According to the World Health Organization, there were 1.8 million new CRC cases diagnosed globally in 2018, with 862,000 deaths attributed to CRC (6). The public health concerns associated with CRC are more serious due to its growing prevalence worldwide. This underscores the pressing need to enhance preventive, early detection, and therapeutic approaches to mitigate their adverse impact on global health and socioeconomic circumstances (7). However, conventional treatments for CRC have demonstrated slight effectiveness, underscoring the pressing need for novel therapeutic strategies.

Some species of helminths have been proposed as potential treatments for diseases such as inflammatory bowel disease, celiac disease, atherosclerosis, non-alcoholic fatty liver disease, and multiple sclerosis (8–12). Many studies have also indicated that helminths can potentially treat cancer (13–17). This review aims to investigate the potential of helminths to enhance risk factors associated with the development of CRC, prevent the occurrence of CRC, and analyze the correlation between helminths and the development and prognosis of CRC.

## 2 Factors associated with colorectal cancer

### 2.1 Precursors of colorectal cancer

**Figure 1:**
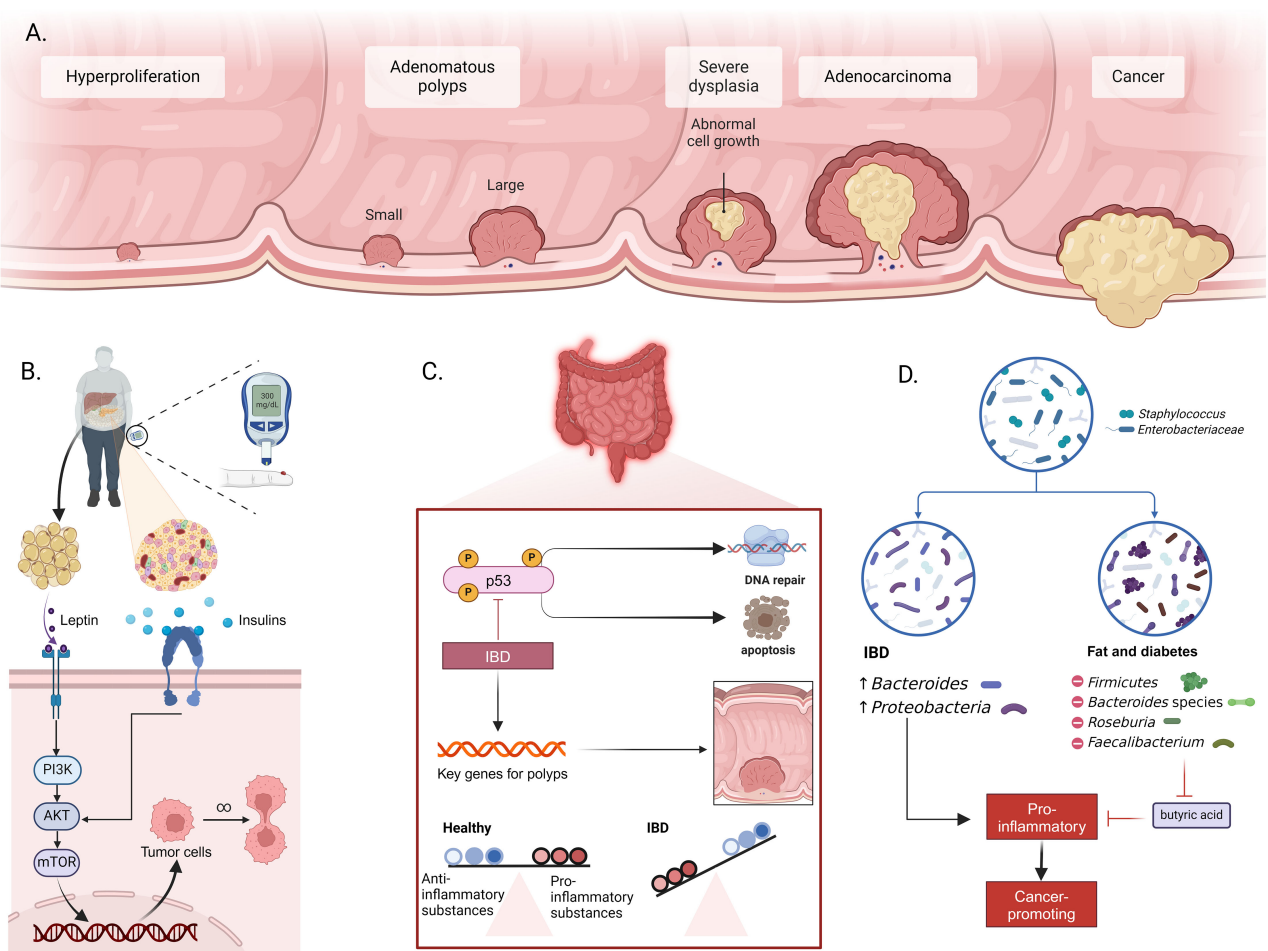
Mechanisms of intestinal inflammation, metabolic dysfunction, and CRC development. **(A)** Progression from normal epithelium to CRC, including hyperplasia, formation of small and large adenomatous polyps, progression to severe dysplasia, adenocarcinoma, and ultimately cancer. **(B)** Role of metabolic dysfunction in CRC. Adipose tissue releases leptin and insulin, activating the PI3K/AKT/mTOR signaling pathway in intestinal cells. This activation promotes tumor cell survival and proliferation, leading to CRC development. **(C)** Impact of inflammatory bowel disease (IBD) on CRC incidence. Inflammation induces activation of p53 and other key genes related to polyp formation. Chronic inflammation disrupts the balance between proinflammatory and anti-inflammatory substances, promoting DNA damage and resistance to apoptosis. **(D)** Changes in gut microbiota associated with IBD and metabolic diseases. IBD is associated with increased Bacteroides and Proteobacteria, while metabolic diseases like obesity and diabetes are linked to changes in Firmicutes, Bacteroides, Roseburia, and Faecalibacterium populations. These changes enhance pro-inflammatory conditions, reduce butyrate production, and facilitate the formation of a cancer-prone environment.

Specific genetic mutations in oncogenes, tumor suppressor genes, and genes associated with DNA repair pathways are the cause of CRC. It starts from polyps and abnormal crypts, progresses to early adenomas, then to advanced adenomas (**Figure 1A**), and eventually results in CRC. About 70% of CRC cases follow this mutation sequence. While genetic and environmental factors both influence CRC development, most cases are sporadic, with only around 25% having a family history, indicating that acquired factors are key contributors (18).

Obesity and diabetes significantly increase CRC risk. Obesity, linked to leptin production by adipose tissue, activates macrophage growth, migration, and cytokine production and stimulates multiple signaling pathways such as JAKs/STATs and PI3K/AKT, which promote cancer cell proliferation and metastasis (**Figure 1B**) (19, 20). Similarly, diabetes accelerates CRC development via insulin resistance and IGF system alterations, with elevated IGF-I levels promoting cell proliferation and cancer growth (21).

Patients with IBD have a 2–6 times higher CRC risk due to complex carcinogenic mechanisms (22). Inflammation causes oxidative stress and DNA damage, triggering a sequence from inflammation to dysplasia to cancer (**Figure 1C**) (23, 24). This process activates oncogenes and deactivates tumor suppressor genes like p53 (25, 26). IBD also induces chromosomal instability and disrupts intestinal ecology, promoting CRC through the production of carcinogens and the breakdown of epithelial barriers (27, 28). The indirect carcinogenic pathway involves cytokines released by inflammatory and epithelial cells, with IL-6 playing a crucial role in CRC pathogenesis through JAK-STAT3 activation. Tumor-associated macrophages and leukocytes further drive chronic inflammation and carcinogenesis (29, 30). In IBD patients, these mechanisms significantly increase CRC risk, highlighting the importance of early diagnosis and effective treatment to reduce the risk of progression.

Alterations in gut microbiota composition also contribute to CRC risk. IBD and metabolic diseases disrupt the microbial balance, increasing CRC incidence. IBD patients have a lower microbial gene count, with Bacteroides and Proteobacteria increased, while healthy individuals predominantly have Actinobacteria and Verrucomicrobia (**Figure 1D**) (31). In obese patients, Firmicutes increase, while Bacteroides decrease, leading to increased intestinal permeability and inflammation. This dysbiosis promotes the transition from adenoma to invasive cancer. The loss of butyrate-producing bacteria in obesity and type 2 diabetes further promotes inflammation and tumorigenesis (32, 33).

Moreover, reduced microbial diversity is strongly linked to CRC development, as it activates NF-κB signaling and triggers inflammatory processes, which contribute to carcinogenesis (34, 35). Therefore, regulating microbial composition may offer a promising approach to CRC prevention and treatment.

### 2.2 The role of cytokines in the development and prognosis of colorectal cancer

Specific cytokines, such as FOXP3, TNF-α, and IFN-γ, regulate tumor immunity, often with elevated expression in CRC. FOXP3 activation in cancer cells leads to cytokine secretion (TGF-β, IL-10), suppressing immunity, and is linked to poor prognosis (36–38). TNF-α, produced by macrophages, promotes epithelial-mesenchymal transition, aiding metastasis; high TNF-α expression predicts tumor deterioration (39, 40). IFN-γ activates macrophages, with its deficiency promoting CRC development. In contrast, specific IFN-γ expression enhances innate immunity and suppresses tumors, correlating with better prognosis (41–43).

Several interleukins contribute to CRC progression. IL-1β, a pro-inflammatory cytokine, boosts cell proliferation and increases CRC risk (44, 45). IL-17 from CD4+ T cells supports tumor development and angiogenesis by stimulating VEGF production in cancer cells (46, 47). Elevated levels of IFN-γ, IL-12, IL-15, and IL-18 are linked to favorable CRC outcomes, while IL-4, IL-6, IL-17, TNF, TGF-β, and VEGF indicate tumor progression (48–52).

## 3 Anticancer mechanisms of helminths

The intricate and significant subject of helminth infection’s influence on tumor growth. Some helminth infections are indeed associated with cancer, such as *clonorchiasis* and *Opisthorchis viverrini* (pathogens of cholangiocarcinoma) and *Schistosoma japonicum* (a risk factor for liver and CRC) (53, 54). The regulation of immune responses by parasitic infections is also an important aspect. For instance, the generated Th1 immune response can suppress tumor growth and be essential in the early stages of *Eimeria granulosa* infection to eliminate cancer cells (55, 56); however, as the infection progresses, the activation of the Th2 immune response may promote tumor progression and metastasis. This phase-dependent change in immune response requires further research to understand its impact on tumor development (57, 58). Additionally, the anti-cancer activity induced by parasitic infection is limited by the virulence of the live parasite and the morbidity induced. Although attempts to use live vaccination strategies have garnered significant attention, their effectiveness is not perfect. Therefore, consideration is being given to using parasite-derived products as new therapeutic agents (59–61).

### 3.1 Helminth-mediated immune cells and immune factors

Helminth infections often mediate immune responses that lean towards immunosuppression. For example, through a TGF-β-dependent mechanism, the excretory/secretory (E/S) products of *Echinococcus multilocularis (E. multilocularis)* larvae induce the transformation of CD4(+) T cells into Foxp3(+) Tregs *in vitro*. When T cells come into contact with E/S products, they release more of the immunosuppressive cytokine IL-10 (62). Helminth-generated extracellular vesicles (EVs), similar to exosomes, also play a crucial role in parasite-host interactions. For example, EVs from *Nippostrongylus brasiliensis* protect mice from intestinal inflammation when administered via a single intraperitoneal injection. Important inflammatory cytokines linked to colitis pathophysiology, including IL-6, IL-1β, IFNγ, and IL-17a, were markedly suppressed in the colon tissues of mice given EVs (63).

**Figure 2:**
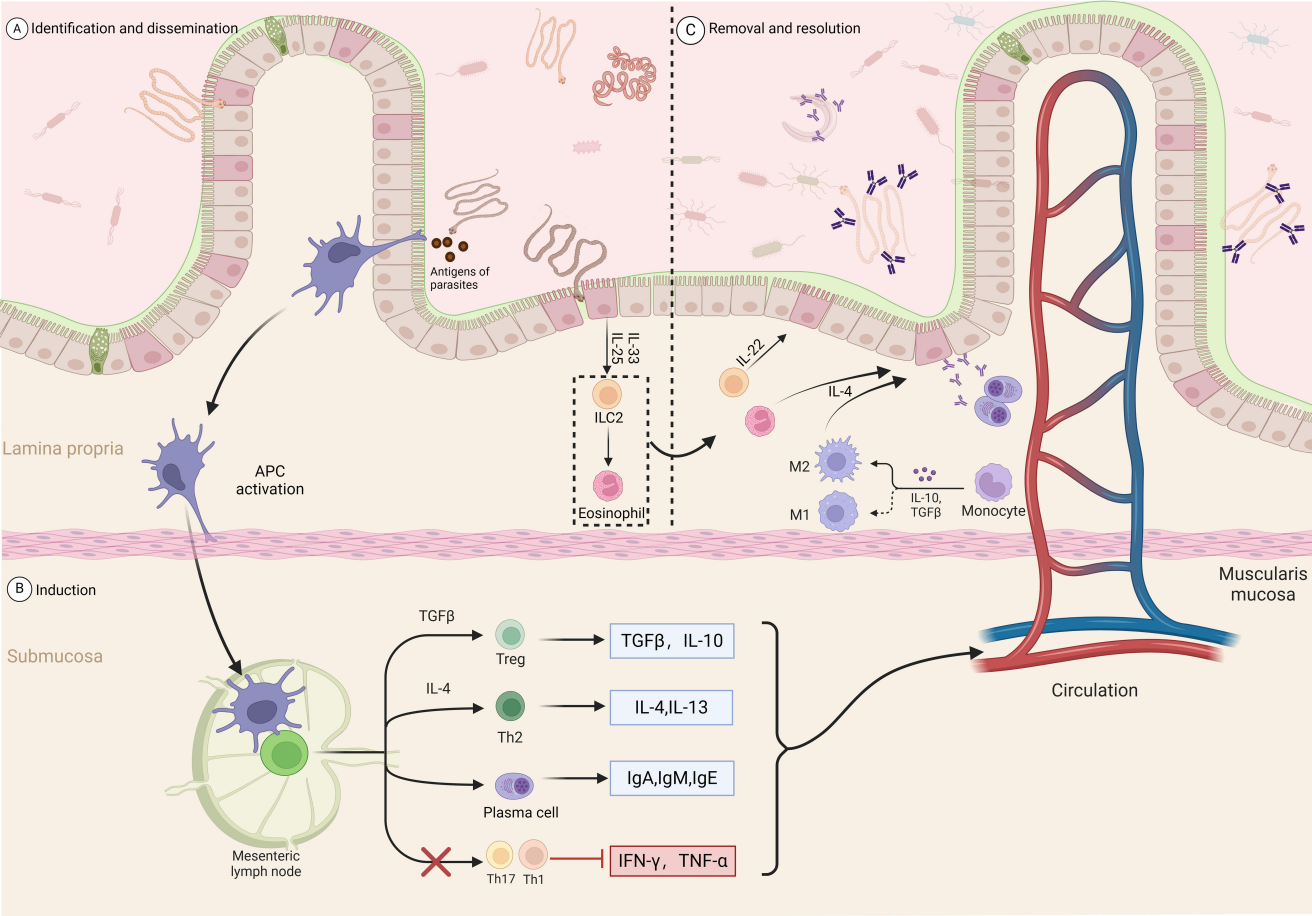
Intestinal immune response process induced by parasitic infection. Recognition and dissemination: **(A)** Parasitic antigens are recognized and captured by antigen-presenting cells (APCs) in the lamina propria. **(B)** Induction: APCs activate T cells in the mesenteric lymph nodes. TGF-β and IL-10 promote the differentiation of regulatory T cells (Tregs), while IL-4 promotes the differentiation of Th2 cells. Th2 cells secrete IL-4 and IL-13, further stimulating plasma cells to produce IgA, IgM, and IgE antibodies. Meanwhile, Th17 and Th1 cell responses are inhibited, reducing the production of IFN-γ and TNF-α. **(C)** Clearance and resolution: In the lamina propria, ILC2 cells secrete IL-4, IL-13, and IL-22, recruiting and activating eosinophils and M2 macrophages to promote anti-parasitic immune responses. Monocytes also participate in the resolution of inflammation under the influence of IL-10 and TGF-β. Through multiple pathways, the inflammatory response is regulated, restoring intestinal homeostasis.

CD4+ T cell subsets play a key role in the host’s protective response against expelling helminths while also regulating many inflammation and immune parameters associated with parasite expulsion (64) Th1 cells facilitate the control of intracellular infections by generating cytokines such as IFN-γ, IL-2, and IL-12. However, in most cases, the host’s immune response to helminth infections is primarily driven by Th2-like T helper cell responses, significantly producing IL-4, IL-5, IL-10, IL-13, IL-25, and IL-31 (65–68), to facilitate protective settlement against helminths (69). Epithelial cells are among the first cell types to encounter intestinal helminths (70). Therefore, when stimulated, damaged, or dying, they release cytokines, including IL-25 and IL-33, which are crucial in inducing innate immune responses and promoting type 2 inflammation processes (71). The interaction of these immune factors leads to the further development of immune responses, including the recruitment of eosinophils, B cells, and M2 macrophage activation (72); helminth infections can also increase mucus production by intestinal goblet cells, promoting beneficial bacterial growth; inhibiting harmful bacteria, and exerting anti-inflammatory effects (**Figure 2**) (73, 74).

### 3.2 Helminth-derived compounds

#### 3.2.1 Antigen activation targeted immunotherapy

Current cancer immunotherapy strategies aim to stimulate host immune cells to target tumor-associated antigens (TAAs), either directly or indirectly, to attack cancer cells (75). Vaccination remains a promising and significant approach to cancer immunotherapy. However, clinical barriers, such as immune tolerance to TAAs, limited immunogenicity of TAAs, and active immune evasion mechanisms employed by advanced cancers, hinder its success (76). Overcoming these barriers may require non-toxic immune modulators or adjuvants to boost both innate and adaptive tumor-specific immune responses, forming the basis for successful vaccine formulations (77).Parasite - derived antigens may hold the key to overcoming these obstacles and improving the effectiveness of cancer vaccines.

Although a variety of tumor-specific antigens have been identified and utilized, most do not trigger strong and appropriate immune responses (78). Tumor - associated glycoproteins, such as sialyl - Tn, TF, and Tn, are often used in cancer diagnostics and prognosis and are mainly found on cancer cells (79). These glycoproteins have also been found in the adult and larval stages of Schistosoma mansoni and in patients with cystic echinococcosis (CE) (80). There is evidence that hydatid cyst antigens have anticancer effects against various types of cancer *in vitro* and mouse models, including CRC (81–84). Some anti-parasitic drugs can inhibit tumor growth, suggesting that there may be shared antigenic epitopes between certain parasites and cancer cells (85). Given the presence of these antigens in both parasites and cancer cells, it is not surprising that there is an immunological cross-reactivity between the two. Tn and sTn antigens linked to cancer have been identified in both the larval and adult stages of *E.granulosus* (80), as well as TF antigens linked to CRC (86). It has been confirmed that there is immunological cross-reactivity between the sera of cancer patients and parasite antigens (87), which means that the host immune system can recognize these common antigens and attack them. While antigens from cancer cells usually have weak immunogenicity, those from microbes often have strong immunogenicity (88, 89). Therefore, parasite - derived antigens offer a way to overcome the immunogenicity limitations of cancer antigens and may enhance immune responses against cancer cells (81). Immunizing with parasite antigens could change the immunosuppressive tumor microenvironment into an immune-supportive one, amplifying anticancer immune responses and opening up new possibilities for CRC treatments.

In the exploration of prospective vaccine targets, numerous significant antigens/proteins from helminths have demonstrated considerable promise. *Heligmosomoides polygyrus* antigens modulate macrophage activities, enhancing immune regulation within the tumor microenvironment and inhibiting breast cancer cell growth (90). Likewise, the *E. granulosus* antigen B component promotes M1/M2 macrophage polarization, hence augmenting inflammation and facilitating pathogen clearance (91). Specific glycans generated from parasites, including IL-33 modulators, enhance the activation of antigen-presenting cells such as dendritic cells, highlighting their potential in cancer immunotherapy (92). *E. granulosus* antigens, in both crude hydatid cyst forms and Tn-like peptides, elicit significant IFN-γ production in immunized mice, signifying Th1 polarization (93). Antigens from Trypanosoma cruzi, employed in CRC-specific anticancer vaccines, have demonstrated considerable efficacy in inhibiting tumor growth in the colon in many investigations (13, 15). Additionally, a new recombinant protein from Toxoplasma gondii, rGRA6Nt, has demonstrated the ability to limit tumor growth in murine colorectal cancer models while simultaneously tripling the density of CD8+ T cells within the tumors (94). These findings highlight the revolutionary potential of parasite antigens in the advancement of successful cancer immunotherapies.

Parasite antigens, sharing immunomodulatory pathways with viral antigens, have demonstrated the ability to upregulate immune-activating genes and enhance T cell activity in HTLV-1 virus models, echoing mechanisms observed in tumor immunotherapy (95). These antigens also exhibit potential in managing immune toxicities, such as CAR-T therapy-related neurotoxic syndromes (96). Their broad immunological and biological activities position them as valuable tools for the next generation of cancer immunotherapy.

## 4 CRC treatment strategies

### 4.1 Adverse outcomes of live helminth strategies

The regulation of CRC immune factors by helminth infections is a complex process worthy of in-depth exploration. Changes in cytokines can reflect the impact of helminth infections on the immune system, where pro-carcinogenic factors such as IL-10, TGF-β, and IL-35 show an increased risk, while anti-carcinogenic factors like IL-6, IL-1β, IFNγ, and IL-17a exhibit a downward trend. This phenomenon suggests that helminth infections may primarily promote carcinogenesis. *The* International Agency for Research on Cancer has categorized *Schistosoma haematobium (S. haematobium)* as a Group 1 carcinogen (97), closely associated with the incidence of bladder squamous cell carcinoma. *S. mansoni*, on the other hand, is classified as Group 3, with insufficient evidence currently confirming its carcinogenicity in humans. Nonetheless, new findings from studies using cell cultures and animal models indicate that *S. haematobium* may promote the development of liver and CRC (98). Recent studies also indicate that certain parasitic infections can lead to downregulation of tumor suppressor genes; for instance, *Theileria annulata* infection promotes p53 suppression and genomic instability (99). Similarly, *Toxocara canis (T. canis)*infection may create an immunosuppressive tumor microenvironment, increasing tumor size and weight and potentially increasing the risk of breast cancer by reducing P53 gene expression (100–102). The evidence linking helminth infections to carcinogenesis is substantial (**Table 1**), suggesting that despite various interacting factors contributing to CRC, direct use of live helminth infections as a therapeutic agent for CRC is not advisable. Without sufficient clinical trial support, we cannot definitively determine how helminth infections affect immune responses. Therefore, a more profound understanding of the mechanisms of helminth infections is needed to develop more effective strategies for CRC prevention and treatment.

**Table 1:** Exacerbation of cancer due to helminth and protozoan parasite infections.

Parasite Type	Scientific Name	Immunity		Conclusion	Ref
		Increase	Decrease		
Protozoan Parasite	*Toxoplasma gondii* *(T. gondii)*	ND	ND	*T. gondii* is a significant factor in primary intraocular B-cell lymphoma.	(103)
Helminth	*Echinococcus* *granulosus* *(E. granulosus)*	CD25+ T cells CD4+ T cells	Th1 cells	The presence of *E. granulosus* infection and breast tumors can significantly increase the risk of cancer metastasis.	(104)
Protozoan Parasite	*Cryptosporidium* *Parvum* *(C. parvum)*	CD4+ T cells CD8+ T cells	ND	Immunosuppressive conditions can lead to cancer development and metastasis by promoting chronic inflammation and overexpression of cyclin D1.	(105, 106)
Protozoan Parasite	*C. parvum*	P53tumor suppressor labelings	ND	Cryptosporidium has been linked to the development of gastrointestinal cancers.	(107)
Helminth	*Strongyloides* *stercoralis*	Neutrophils, eosinophils	ND	*Strongyloides stercoralis* infection is comorbid with gastric adenocarcinoma, with larvae present in abnormal glands.	(108)
Helminth	*Fasciola gigantica*	TNF-α IL-1β IL-6	ND	The carcinogenic process induced by *Fasciola gigantica* infection is significantly influenced by oxidative stress and free radicals produced by inflammatory cells.	(109)
Protozoan Parasite	*Plasmodium* *falciparun*	ND	ND	Burkitt lymphoma is linked to prolonged and severe malaria exposure.	(110)
Protozoan Parasite	*Theileria*	ROS	ND	The parasite's transformation involves reprogramming glucose metabolism and redox signaling, revealing its molecular strategies for causing cancer-like phenotypes in host cells.	(111, 112)
Protozoan Parasite	*Trichomonas* *vaginalis*	IL-8	ND	The release of TvMIF during *Trichomonas vaginalis* infection has been linked to the potential promotion of prostate cancer progression.	(113)
Helminth	*Heligmosomoides* *polygyrus*	IL-10 IL-4 IL-13	ND	Intestinal helminth infections can lead to the development and progression of colitis-associated CRC by affecting the immune response.	(114)
Helminth	*Trichuris muris*	IL-10, Treg cells	ND	The intestine experiences enhanced tumor alterations and progression.	(115)
Helminth	*Blastocystis* sp.	ND	ND	CRC patients exhibited a significantly higher prevalence of *Blastocystis* sp. than the control group.	(116)
Helminth	*Clonorchis sinensis*	TNF-α IL-1β IL-6	ND	*Clonorchis sinensis i*nfection causes DNA damage in the biliary epithelium, disrupting homeostatic mechanisms and leading to malignant transformation.	(117)

### 4.2 Relieve precursors

When exploring the impact of helminth infections on immune regulation in CRC, it is essential to consider the risk factors in epidemiology and think about how to mitigate them to prevent CRC. Inflammation is a common characteristic among these risk factors. Therefore, controlling inflammatory immune responses becomes a key strategy in reducing risk.

Helminth infections positively impact the treatment of IBD (118, 119). Research primarily focuses on two regulatory factors: IL-10 and TGF-β (120–122) IL-10 and TGF-β can inhibit the production of inflammatory mediators. Engineered lactobacilli expressing IL-10 successfully prevented the development of colitis, showing potential as a treatment for IBD (123, 124). Studies have confirmed that *Heligmosomoides polygyrus (H. polygyrus)* can relieve colitis symptoms in animals lacking IL-10 (125) and that schistosome eggs protect mice against colitis produced by TNBS (126). Increasing TGF-β activity has also shown potential value in treating inflammatory bowel disease (127, 128). Helminth infections can induce the production of the Th2 cytokine IL-4, further activating and driving the output of TGF-β (129, 130). This plays a key role in inhibiting the autoreactive Th1 and Th17 responses that mediate autoimmune diseases (131).

A meta-analysis showed that some parasitic infections benefit human metabolism, such as lowering fasting blood glucose and HbA1c levels and reducing the prevalence of metabolic syndrome and type 2 diabetes (132). In managing obesity and diabetes, studies have found that various parasitic helminths positively impact the immune response to obesity or malnutrition by regulating Th2 immune responses (133). Helminth infections significantly reduced insulin resistance, liver fat accumulation, and fatty acid synthase gene expression in mice, possibly due to the upregulation of Th2 factors promoting the production of alternatively activated macrophages, which secrete IL-10 to inhibit inflammation (134). Research also indicates that Omega-1, a substance from *Schistosoma mansoni(S. mansoni)* eggs, effectively induces Th2 cell responses in mice and improves obesity caused by a high-fat diet (135–137). Studies have found that various soil-transmitted helminths, such as *Ascaris* spp and *Ancylostoma duodenale*, can improve BMI, enhance tissue insulin sensitivity, and reduce the risk of metabolic syndrome (138). Additionally, mice infected with *H. polygyrus* and their offspring showed significantly reduced weight gain on a high-fat diet, possibly due to changes in gut microbiota and increased short-chain fatty acid levels (139). These results suggest that helminth infections not only inhibit obesity but also potentially positively affect the treatment of diabetes (140).

The preservation of human health depends on gut bacteria (141). The interaction between parasites and the gut microbiota also significantly impacts host health (142). The gut microbiome’s diversity and abundance change when helminths are present (143). According to studies, mice infected with nematodes that resemble hookworms had far lower fasting blood glucose levels, with a marked increase in *Lactobacillus* in their gut microbiota (144), a genus known for its benefits in T2D (145). Research has also found that helminth infections can indirectly regulate NE concentration to inhibit obesity by altering the gut microbiota (146). Because the gut microbiotas of mice and humans differ significantly, more research is required to determine the precise effects of helminths on the gut microbiota, even though these findings highlight the positive impact of helminths on the host gut microbiota, particularly in improving metabolic health and resisting metabolic-related diseases.

### 4.3 Helminth-derived compounds may become therapies for colorectal cancer: increasing evidence supports this

Although helminth infections may promote cancer development, certain derivatives of these helminths show anticancer potential. For example, high-pressure sterilized *S.mansoni* antigens have exhibited anticancer protective effects in DMH-induced Colorectal cancer mice (147). Similarly, antigens from *T.canis* can stimulate immune responses and suppress cancer cells at high concentrations (148). It has been demonstrated that *T.canis* extracts stimulate human leukocytes to produce Th1 and regulatory cytokines. Their secreted proteins are highly similar to anticancer drugs in computer analyses (148). Additionally, antigens from *E.granulosus* have demonstrated anticancer effects *in vitro* and animal models; for instance, EgKI-1 can induce anticancer effects in tumor tissues, suggesting potential development as a cancer vaccine (78). Recent studies have further shown that molecules derived from the helminth *Taenia crassiceps* (*T. crassiceps*) can enhance the effectiveness of the chemotherapeutic agent 5-fluorouracil (5FU) in treating CRC. *T. crassiceps* modulates inflammatory cytokines, alters the tumor microenvironment, and promotes the recruitment of immune cells such as NK cells and CD8+ T cells, thereby increasing tumor cell apoptosis and reducing tumor growth (149).

Research on helminth-derived compounds for anticancer purposes is not limited to these examples alone. Many *in vitro* and animal experiments have shown that helminth derivatives have inhibitory effects on cancer, including studies on CRC (**Table 2**). These findings provide new perspectives on the complex relationship between helminth infections and cancer and open new possibilities for developing therapeutic strategies based on helminth-derived compounds.

**Table 2:** Potential of helminth and protozoan-derived products in cancer treatment.

Parasite Type	Scientific Name	Antigen/pr otein names	Trial Category	Target cancer	Result	Conclusion	Ref
Protozoan Parasite	*Toxoplasma gondii* *(T. gondii)*	ATV	Animal experiment	Ehrlich solid carcinoma (ESC)	**Tumor Metrics**: 13.3% of the incidence of ESC was inhibited by ATV. A marked decrease in the volume and weight of the tumors in mice given the ATV vaccination. **Immunological Response**: In ESC, there are more CD8+ T cells and fewer FOXP3+ Treg cells, which have a considerable antiangiogenic function and an elevated CD8+/Treg ratio.	*Toxoplasma* vaccination significantly prevented ESC.	(150)
Protozoan Parasite	*T. gondii*	CPS	Animal experiment	B16F10 melanoma	**Tumor Metrics**: Within 12 days, all of the treated mice's tumors had stopped growing and had swiftly shrunk, becoming undetectable. The mice received CPS treatment. **Immunological Response**: Tumor-specific CD8+ T cells and IFN-γ production increased significantly as a result of the treatment, suggesting a robust immune response.	Attenuated *T. gondii* induced strong anticancer immune responses.	(151)
Protozoan Parasite	*T. gondii*	CP	Animal experiment	ESC	**Tumor Inhibition**: According to the study, when compared to the ESC control group, all treatments caused noticeably higher necrosis in the tumor cells. **Immunological Response:** According to immunohistochemistry analysis, CP therapy increased the number of CD8+ T cells and decreased the number of Treg cells, raising the CD8+/Treg cell ratio surrounding the tumor.	Inactivated *T. gondii* vaccine suppressed ESC growth through immune modulation.	(152)
Protozoan Parasite	*T. gondii*	Radiation- attenuated vaccine	Animal experiment	Ehrlich ascites carcinoma	**Immunological Response:** By inducing long-lasting immunity, activating interferon γ, and downregulating transforming growth factor β, the vaccination effectively inhibited the growth of tumors. Additionally, it markedly reduced the content of nitric oxide, angiogenic factors (VEGF-A, integrin, MMP-2, and MMP-9), and tumor-promoting inflammatory markers (TNF-α and STAT-3).	Effective in immune activation and tumor targeting, usable as a preventive or adjunct to chemotherapy.	(153)
Protozoan Parasite	*Trypanos* *oma cruzi* *(T. cruzi)*	Epimastigo te lysates	Animal experiment	CRC, breast cancer	**Tumor Metrics**: Colon and breast cancer models showed a substantial reduction in tumor growth following vaccination with *T. cruzi* epimastigote lysates. **Immunological Response**: Both CD4+ and CD8+ T cells were activated in immunized rats, and their spleens responded more cytotoxically to tumors than did the controls.	Dramatically suppressed tumor growth in rat models of colon and breast cancer by inducing strong antitumor responses (cellular and humoral immunity).	(13)
Protozoan Parasite	*T. cruzi*	rTcCalr	*In vitro*	breast cancer	**Tumor Inhibition:** rTcCalr binds to scavenger receptors, reducing the formation of new blood vessels in the tumor, thereby decreasing the supply of nutrients and oxygen essential for tumor growth and metastasis​​. **Immunological Response**: rTcCalr increases the expression of MHC I while decreasing the expression of MHC II, which helps enhance the recognition and attack of tumor cells by CD8+ T cells​​.	Inhibited tumor growth and enhanced immunogenicity *in vitro*, suggesting potential as a novel cancer therapeutic by modulating membrane molecules.	(154)
Protozoan Parasite	*T. cruzi*	gp82	*In vitro*	melanoma cells	**Tumor Metrics**: According to *in-vivo* studies, C57BL/6 mice treated with J18 at the site of tumor cell inoculation and inoculated with Tm5 cells formed tumors smaller than those developed by mice treated with GST or phosphate-buffered saline, and they also lived longer.	Induced apoptosis in melanoma cells.	(155)
Protozoan Parasite	*T. cruzi*	rP21	*In vitro*	triple- negative breast cancer	**Tumor Metrics**: Treatment with rP21 inhibited the expression of CXCR4, a receptor that is overexpressed in breast cancer cells and other tumor cells, and caused the receptor to internalize.	Inhibited invasion, migration, and proliferation of triple-negative breast cancer cells by downregulating MMP-9 and CXCR4, implying use in treating invasive breast cancer.	(14)
Protozoan Parasite	*T. cruzi*	Epimastigo te lysates	Animal experiment	CRC, breast cancer	**Tumor Inhibition**: Rats immunized with *T. cruzi* epimastigote lysate showed significant suppression of tumor growth in models of colon and breast cancer **Immunological Response**: Both CD4+ and CD8+ T cells were activated in immunized rats, and their spleens responded more cytotoxically to tumors than did the controls.	Elicited potent antitumor immune responses by stimulating cellular and humoral components, suggesting potential for innovative anticancer treatments.	(15)
Helminth	*Trichinell* *a* sp*iralis* *(T.spirali)*	TPD52	*In vitro*	Osteosarcoma	**Tumor Inhibition**: Both *in vivo* and *in vitro*, osteosarcoma cells were driven to undergo apoptosis by anti-TPD52 antiserum. **Immunological Response** It was shown that injecting anti-TPD52 antiserum into nude mice increased their serum levels of TNF-α, IL-12, and IFN-γ using an enzyme-linked immunosorbent assay.	Induced apoptosis without histopathological damage, showing anti-osteosarcoma effects.	(16)
Helminth	*T.spiralis*	ML ESPs	*In vitro*	Lung cancer	**Tumor Inhibition**: The expression of pro-apoptosis genes Bax, Cyt-C, Apaf-1, caspase-9, and caspase-3 was increased when comparing the Western blotting results to the negative control group, while the expression of anti-apoptosis genes Bcl-2 and Livin was decreased.	Caused H446 cells to undergo apoptosis via a mitochondrial pathway, indicating a potential antineoplastic mechanism.	(17)
Helminth	*T.spiralis*	ESP	*Animal* *experiment*	*H22 tumor* *cells*	**Tumor Inhibition**: ESP has the potential to inhibit H22 cell division and initiate apoptosis through the mitochondrial pathway, both *in vitro* and *in vivo* .**Immunological Response** Th1 cytokine levels rose significantly early in the *T.* sp*iralis* infection and had antitumor effects; Th2 cytokines increased later than Th1 cytokines.	Directly triggered tumor cell death and indirectly suppressed proliferation through the host immune system.	(156, 157)
Helminth	*S.mansoni* *T.spiralis*	*Autoclaved* *antigen*	*Animal* *experiment*	*CRC*	**Tumor Inhibition**: Only the harmful effects of DMH-induced colon carcinogenesis were protected against by *S.mansoni* antigen administration, which also resulted in a notable reduction in the average lesion size and number of neoplasias per animal. **Immunological Response:** a noteworthy drop in serum IL-17, a noteworthy rise in serum IL-10, and a notable proportion of intestinal FoxP3+ Treg cells and splenic CD4+T cells.	Autoclaved antigen reduced lesion size and neoplasia number, protecting against DMH-induced colon carcinogenesis.	(147)

## 5 Future directions

### 5.1 Combination therapy of helminth therapy with conventional treatments

**Figure 3:**
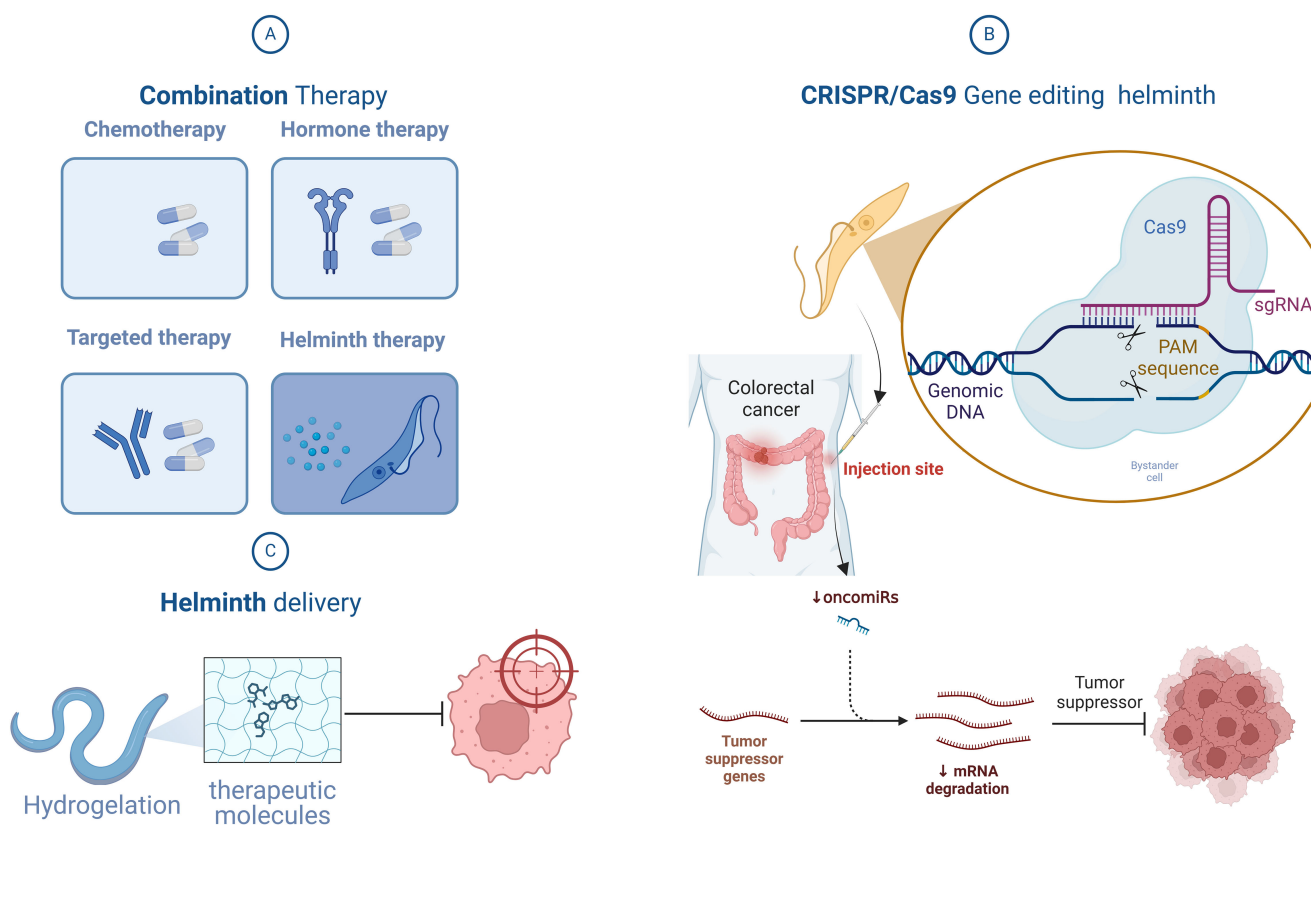
Overview of helminth-based colorectal cancer (CRC) therapy approaches. **(A)** Combination Therapy: Integration of helminth therapy with conventional cancer treatments (chemotherapy, hormone therapy, and targeted therapy) enhances therapeutic outcomes. Helminth therapy modulates the tumor microenvironment, improves immune responses, and increases the sensitivity of cancer cells to cytotoxic treatments. **(B)** CRISPR/Cas9 Gene Editing and miRNA Modulation: Engineering helminths via CRISPR/Cas9 enables precise genetic modifications, such as reducing carcinogenic risks and tailoring parasites for therapeutic purposes. This technology enhances the safety of live vaccines and improves their anti-cancer effects. Additionally, helminth-derived molecules and attenuated parasites can regulate host miRNAs, particularly oncomiRs like miR-21, restoring tumor suppressor gene expression and reducing tumor progression. **(C)** Helminth Delivery System: Nematodes, such as Anisakis simplex, are coated with hydrophilic polymers to bypass immune detection, enabling targeted delivery of therapeutic molecules to tumors. This novel delivery system shows promise for improving drug precision and effectiveness.

Helminth therapy may become an option for the prevention and treatment of CRC. Despite some helminths having carcinogenic potential, antigens derived from helminths are being studied for their potent anti-cancer activity while minimizing side effects as much as possible (93). To generate helminth antigens at high concentrations, researchers should concentrate on creating standardized and controlled helminth antigen compounds by carefully identifying and extracting crucial immunogenic components (158). Antigen composition and structure can be precisely manipulated to increase antigen safety and effectiveness. Engineered recombinant antigens allow for fusion and modification to achieve maximum anti-cancer efficacy. These antigen characteristics include enhancing immunity, reducing side effects, or improving tissue targeting (159). When used in combination with conventional therapies, helminth therapy may produce unexpected results. Immune system-focused treatments, including immune checkpoint inhibitors or adoptive cell therapy, may benefit more from helminth-induced immune regulation (**Figure 3A**) (160).

Depending on the stage of cancer, for inflammatory cancers, helminth therapy can enhance the regulatory and anti-inflammatory milieu it induces, hence improving immune responses to malignancies (161). Additionally, helminth therapy may sensitize cancer cells to chemotherapy, making them more susceptible to the cytotoxic effects of chemotherapy. Helminth therapy can reduce the amount of time and dose required for treatment while increasing the effectiveness of chemotherapy by altering the tumor microenvironment and boosting immune responses (162).

### 5.2 Gene editing reduces the toxicity of the helminth

Live parasitic infections may exacerbate symptoms of CRC and other cancers; hence, utilizing gene editing technology is a promising approach when considering live vaccines. With the rapid advancement of biotechnology, next-generation CRISPR gene editing technologies such as CRISPR-Cas13 (163) can be employed to engineer parasites with specific traits: short lifecycle, inability to proliferate, or sensitivity to particular drugs. Recent genetic engineering tools like CRISPR/Cas9 allow for gene knockout/deletion in parasitic helminths, with potential for future exploration in gene knock-in/insertion within parasite genomes (**Figure 3B**) (164). Current research indicates that CRISPR/Cas9 gene editing can enhance the safety of parasite eggs (165), particularly those of blood flukes, affirming the feasibility of developing and applying *S. mansoni*, a carcinogenic parasite, for CRC therapy. CRISPR and CRISPR-based alternative technologies will continue to thrive, potentially aiding the development of new strategies involving parasite antigens to tame indigenous parasitic helminths (166), thereby integrating more carcinogenic parasites into helminth therapy shortly.

### 5.3 Regulating oncomiRs therapy

A class of molecules known as small non-coding RNAs (miRNAs) that regulate gene expression (167) is thought to be important for cancer treatment. They participate in tumorigenesis by modulating signaling pathways critical for processes such as proliferation, apoptosis, and migration of cancer cells (168–170). Genome analysis studies have revealed dysregulation of multiple miRNAs in cancer, with those overexpressed targeting tumor suppressor genes and stimulating cell proliferation, angiogenesis, and metastasis termed as oncomiRs (171). miR-21 is a widely reported oncomiR, upregulated in CRC (172, 173), suggesting that modulating its expression levels could be a therapeutic approach to inhibit tumor development.

Parasites also manipulate host immune responses by releasing miRNAs to establish chronic infections (174–176). Some parasites release exosomes containing miRNAs that can be absorbed by host cells and affect gene expression (177), especially taken up by immune cells such as dendritic, macrophage, and monocytes (178). Subsequent enzymatic and protein interaction cascades mediate gene expression in eukaryotes (179). According to related studies, after infection with attenuated *Leishmania donovani*, expression of miR-21 significantly decreases in macrophages and DCs. In contrast, cells infected with wild-type parasites show higher miR-21 expression (**Figure 3B**). We observe that attenuated parasites demonstrate anticancer potential in this regard, whereas untreated parasites not only lack anticancer effects but further promote oncomiR upregulation (180). Therefore, using live attenuated parasite vaccines or other therapeutic approaches may contribute to cancer treatment.

### 5.4 Helminth delivery system

Wildan Mubarok and colleagues have developed an innovative method for cancer treatment using *Anisakis simplex*. These helminths are immersed in a phenol polymer, forming a hydrophilic coating that protects them from the immune system. *In vitro* experiments have demonstrated successful drug delivery and cytotoxicity (**Figure 3C**). This technique is not limited to *Anisakis simplex*; it can also be applied to other nematode species, with functional molecules being replaceable as needed. In the future, nematodes could be used to deliver functional “cargo” to a range of specific targets, playing a significant role in cancer treatment. Utilizing a helminth delivery system for cancer therapy is an innovative and promising research direction (181).

By protecting nematodes from immune system attacks and leveraging their natural affinity for cancer cells and delivery capabilities, this method shows significant potential for improving the precision and effectiveness of anticancer drug delivery (181). However, the hydrogel sheath cannot completely maintain internal pH levels against external influences, meaning that the activity of enzymes and nematodes is still affected by pH fluctuations. Applying this technology to colorectal cancer (CRC) treatment remains a challenge. With further research and multicenter trials, this technique could potentially be used in clinical settings, offering new treatment options for cancer patients.

## 6 Challenges

### 6.1 Clinical safety

As a potential therapeutic approach, helminth therapy shows promise in treating conditions like inflammatory bowel disease. However, its safety concerns also warrant attention. Implanting attenuated live helminth vaccines may pose various safety risks, such as direct induction of CRC, uncontrolled infections, organ migration, and potential adverse reactions. Comprehensive assessment and analysis are necessary when selecting helminths to understand their infectivity and potential side effects on human health, ensuring treatment safety and efficacy (182). Currently, the lack of standardized treatment protocols for helminth therapy increases uncertainty in the treatment process. Establishing standardized treatment protocols is crucial to safeguard the safety and effectiveness of treatment (183). Preclinical research and limited clinical trials are essential for evaluating the safety and efficacy of helminth therapy. Sufficient experimentation and validation are needed to advance helminth therapy development and ensure its safety.

With the advancement of modern medicine and information technology, research on parasitic therapy is gradually deepening. We can utilize modern detection techniques to monitor the real-time effects of live vaccines in patients, such as the Helminth Egg AutoDetection (HEAD) system (184)、Kato-Katz method (185)、Mini-FLOTAC technique (186)、qPCR (187) and other latest monitoring technologies. These enable patients to monitor parasite quantity and activity, ensuring infections remain manageable.

### 6.2 Therapeutic effectiveness

Translating these discoveries from murine models to clinical applications is a formidable undertaking. A significant problem stems from the variety of parasitic worm species and the intricacy of their life cycles. This raises issues regarding safety and tolerance, as the introduction of live helminths or their components may elicit unforeseen side effects, including heightened vulnerability to additional infections (188, 189). Moreover, the optimal dosage or duration of parasite infections required to provide protection in humans remains largely unclear, and apprehensions about the possible adverse effects of these infections have impeded clinical research. The analysis of clinical data on experimental helminth infections is further complicated by significant immunological heterogeneity among diverse genetic backgrounds. Indeed, not all research has shown an effect of helminth infections or deworming on allergic inflammation (190, 191), aligning with the absence of therapeutic benefits of helminth infections in human autoimmune allergic inflammation (192–194).

Consequently, the selection of suitable helminth species and the determination of the ideal therapy dosage and duration continue to be important unresolved issues in scientific research and clinical practice. While proteins produced from helminths exhibit potential in modifying immune responses and mitigating inflammation in conditions such as inflammatory bowel disease and asthma, the exact mechanisms of their actions remain inadequately elucidated (195, 196). The control of pro-inflammatory cytokines and the activation of anti-inflammatory responses have been noted; however, these effects are typically localized and lack consistent replication at the systemic level (196, 197). Moreover, elements such as host genetics, food, and environmental variables influence variability in treatment responses, complicating the uniformity of therapy regimens (61). Species-specific variations among helminths and the development of suitable dosage regimens are critical elements that necessitate more research to enhance treatment results. Similar to other biological therapies, helminthic treatments also pose the danger of resistance development, as the host immune system may progressively establish tolerance, hence diminishing therapeutic efficacy over time (198). The clinical efficacy of helminth therapy may be considerably influenced by the formulation and storage conditions of the therapeutic helminths. Experimental investigations indicate that inappropriate pH levels during storage can diminish the therapeutic efficacy of Trichuris suis eggs (199). The advancement of helminth-derived biopharmaceuticals encounters additional obstacles concerning the pharmacokinetics and molecular characteristics of these substances (200).

### 6.3 Public cognizance and endorsement

The notion of employing parasites as a therapeutic strategy frequently encounters skepticism and apprehension from the public, which constitutes a significant obstacle to the acceptance and implementation of helminth therapy (201). Moreover, cultural and societal conceptions of helminths vary considerably. In Western societies, where personal hygiene and environmental sanitation are emphasized, the concept of putting helminths into the human body is especially difficult to comprehend. This cultural aversion may hinder the acceptability of helminth therapy and its incorporation into conventional medical practice (11, 202).

### 6.4 Regulatory and economic challenges

Helminth therapy encounters substantial regulatory obstacles owing to the intricate and rigorous approval processes for biological therapeutics. Moreover, comprehensive clinical trials are necessary to establish both safety and efficacy, which can be costly and time-consuming (203). Moreover, the economic obstacles linked to the development and commercialization of helminth-based medicines introduce an additional level of complexity. The expenses associated with research, development, and regulatory approval might be too costly, especially for small enterprises or research groups (203).

## 7 Conclusion

CRC continues to be a significant global health concern, requiring the investigation of novel treatment approaches. This review examines multiple facets of helminths in relation to colorectal cancer. Helminth therapy has considerable potential for treating precursor illnesses of colorectal cancer, such as colitis, obesity, diabetes, and changes in gut flora. By inhibiting the immune system’s reaction to helminth invasion, it is possible to decrease cell damage resulting from inflammation and DNA changes, thereby reducing the likelihood of developing precancerous lesions. However, the possible hazards and therapeutic benefits of helminths are highlighted. Helminth infections can exacerbate cancer progression by weakening the immune system, which is a notable risk factor. On the other hand, antigens derived from helminths exhibit encouraging anti-cancer characteristics and have the potential to be employed as novel constituents in vaccines or therapeutic agents.

Gene editing tools, like CRISPR/Cas9, can mitigate the adverse consequences of live helminth infections while preserving their beneficial therapeutic benefits. One potential approach is to explore the use of helminths to modulate oncomiRs associated with colorectal cancer, to mitigate the abnormal expression of tumor suppressor genes. In addition, helminth delivery systems provide a promising therapeutic strategy by utilizing helminths to transport anticancer medications to specific locations in the colon for more efficient treatment of colorectal cancer.

Future research should give priority to the advancement of standardized helminth antigen compounds, with a particular focus on enhancing their immunogenicity and ensuring their safety. Utilizing helminth antigens to stimulate anticancer immune responses can result in immunotargeted therapy, which, when paired with conventional cancer treatments, can yield remarkably potent outcomes. It is essential to develop uniform standards and monitoring systems for the clinical use of helminth treatment. Using sophisticated detection methods to control helminth infections in controlled situations can improve the safety of helminth therapy. We anticipate that future studies will yield additional evidence substantiating the clinical effectiveness of this therapy, thereby providing a cost-effective and efficient treatment alternative for CRC patients globally.
